# Prevalence, virulence factor genes and antibiotic resistance of *Bacillus cereus sensu lato* isolated from dairy farms and traditional dairy products

**DOI:** 10.1186/s12866-017-0975-9

**Published:** 2017-03-14

**Authors:** James Owusu-Kwarteng, Alhassan Wuni, Fortune Akabanda, Kwaku Tano-Debrah, Lene Jespersen

**Affiliations:** 1 Department of Applied Biology, Faculty of Applied Sciences, University for Development Studies, P. O. Box 24,, Navrongo campus, Navrongo, Ghana; 2 Department of Biotechnology, Faculty of Agriculture, University for Development Studies, Nyankpala, Tamale, Ghana; 3 Department of Nutrition and Food Science, Faculty of Science, University of Ghana, Legon, Accra, Ghana; 4 Department of Food Science, University of Copenhagen, Rolighedsvej 26, DK 1958 Frederiksberg C, Denmark

**Keywords:** *Bacillus cereus sensu lato*, Antibiotic resistance, Virulence gene, Enterotoxin, Dairy product, Emetic toxin gene

## Abstract

**Background:**

*B. cereus* are of particular interest in food safety and public health because of their capacity to cause food spoilage and disease through the production of various toxins. The aim of this study was to determine the prevalence, virulence factor genes and antibiotic resistance profile of *B. cereus sensu lato* isolated from cattle grazing soils and dairy products in Ghana. A total of 114 samples made up of 25 soil collected from cattle grazing farm land, 30 raw milk, 28 *nunu* (yoghurt-like product) and 31 *woagashie* (West African soft cheese). Ninety-six *B. cereus sensu lato* isolates from 54 positive samples were screened by PCR for the presence of 8 enterotoxigenic genes (*hblA*, *hblC*, *hblD*, *nheA*, *nheB*, *nheC*, *cytK* and *entFM*), and one emetic gene (*ces*). Phenotypic resistance to 15 antibiotics were also determined for 96 *B. cereus sensu lato* isolates.

**Results:**

About 72% (18 of 25 soil), 47% (14 of 30 raw milk), 35% (10 of 28 nunu) and 39% (12 of 31 woagashi) were positive for *B. cereus sensu lato* with mean counts (log_10_ cfu/g) of 4.2 ± 1.8, 3.3 ± 2.0, 1.8 ± 1.4 and 2.6 ± 1.8 respectively. The distribution of enterotoxigenic genes revealed that 13% (12/96 isolates) harboured all three gene encoding for haemolytic enterotoxin HBL complex genes (*hblA*, *hblC* and *hblD*), 25% (24/96 isolates) possessed no HBL gene, whereas 63% (60/96 isolates) possessed at least one of the three HBL genes. All three genes encoding for non-haemolytic enterotoxin (*nheA*, *nheB* and *nheC*) were detected in 60% (57/96) isolates, 14% (13/96) harboured only one gene, 19% (18/96) whereas 8% possessed none of the NHE genes. The detection rates of *cytk*, *entFM*, and *ces* genes were 75, 67 and 9% respectively. *Bacillus cereus s. l*. isolates were generally resistant to β-lactam antibiotics such as ampicillin (98%), oxacillin (92%), penicillin (100%), amoxicillin (100%), and cefepime (100%) but susceptible to other antibiotics tested.

**Conclusions:**

*Bacillus cereus s. l.* is prevalent in soil, raw milk and dairy products in Ghana. However, loads are at levels considered to be safe for consumption. Various enterotoxin genes associated with virulence of *B. cereus* are widespread among the isolates.

## Background

The *Bacillus cereus* group, also known as *B. cereus sensu lato*, is a species complex which shows high degree of phenotypic and genotypic similarity. The group classically consists of Gram-positive, rod-shaped, spore-forming aerobic bacteria that are widespread in natural environments. The genetic similarity within the *B. cereus* group has been widely studied [1–6].

*Bacillus cereus s. l.* has been found to have significant impact on human health, agriculture, and food processing [5]. *B. cereus* commonly cause spoilage in food products [7]. Additionally, it is an opportunistic pathogen which can cause two types of food poisoning in humans, characterized by either nausea and vomiting or abdominal pain and diarrhea [8, 9].

The virulence of *B. cereus sensu lato* is attributed to different factors. Diarrheal disease is associated with the production of enterotoxins such as hemolysin BL (HBL), non-hemolytic enterotoxin (NHE), cytotoxin K, and enterotoxin FM [10–15], whiles the virulence of emetic strains is attributed to the production of a heat stable cereulide, synthesized by a non-ribosomal peptide synthetase encoded by *ces* genes [16]. The emetic toxin, usually preformed in food, is not inactivated during food processing or gastrointestinal passage because it is highly resistant to heat treatments, extreme pH conditions and protease activities [17–19]. Therefore, ingestion of living *B. cereus* is not necessary for illness of this type to occur. On the other hand, diarrheal food poisoning is not caused by preformed toxins in food, but by viable vegetative *B. cereus* cells (not spores) producing enterotoxins in the small intestine, because spores do not produce enterotoxins. Additionally, spores are easily degraded under gastrointestinal conditions by the host’s digestive enzymes [20–22].

Ghanaian traditional milk products including nunu (yoghurt-like) and woagashie (cheese-like) are produced mainly by spontaneous fermentations of raw cow milk [23, 24]. These products are consumed widely throughout Ghana. However, there has been increasing public concern about the safety of consuming the Ghanaian milk products due to the crude methods of milk production, handling and processing which expose the products to possible contamination with various potential foodborne pathogens such as *B. cereus*. To ensure consumer safety and increase patronage of the Ghanaian traditional dairy products, attempts have been made to characterize the dominant microorganisms and to select starter cultures for the production of quality and safe products [24, 25]. The incidence of *Bacillus cereus* on dairy farms and in milk products has been reported elsewhere, particularly in Scandinavia, the Netherlands, Australia and Brazil [26–29]. However, there is currently no reported study on the prevalence of *Bacillus cereus* in dairy farms and milk products in Ghana, and their associated virulence and antibiotic resistance profile. This study therefore sought to assess the prevalence, virulence factor genes and phenotypic antibiotic resistance of *Bacillus cereus sensu lato* isolated from cattle grazing farms, raw milk, and traditional dairy products in Ghana.

## Methods

### Sampling

A total of 114 samples made up of 25 soil collected from cattle grazing farm land, 30 raw milk, 28 nunu (yoghurt-like product) and 31 woagashie (West African soft cheese) were used for the isolation of *B. cereus.* Raw milk, nunu and woagashie samples were purchased from retail markets in Tamale in the Northern region of Ghana. Raw unpasteurized milk and its products sampled were originally sourced from dairy farms which do not rely on supplementary feeding or routine antibiotics use. There was also no record of *B. cereus* infection among the cattle herds. Soil samples were collected from cattle grazing fields located within 10 mile radius in farming communities in the Northern Region of Ghana. Soil samples were taken from sites at least 500 m apart with a sterilized spatula down to a depth of about 20 cm from the ground surface into sterile stomacher bags and transferred to the laboratory for analysis. Sampling was done between January and October, 2015.

### Isolation of and identification of *B. cereus s. l.*

For the isolation of *Bacillus cereus s. l.*, 25 g of each sample was transferred into 225 ml of sterile phosphate buffered saline (PBS) in a sterile stomacher and homogenized for 2 min using BagMixer stomacher (Inter science, St Nom, France). The homogenate was serially diluted (10-fold) in sterile PBS and 0.1 mL of each dilution was inoculated onto duplicated agar plates containing *B. cereus* agar base (Oxoid, UK) supplemented with 100 ml/l of Egg Yolk Emulsion (Oxoid,) and 5 ml/l of Polymyxin B Selective Supplement (Oxoid). Plates were incubated at 30 °C for 24 h and observed for growth. Suspected *B. cereus* colonies with blue appearance (typically mannitol-negative) and lecithinase positive (zone of precipitation around colonies) were selected from each plate and sub-cultured on nutrient agar (Oxoid). Suspected colonies were further identified by phenotypic and biochemical tests [30] including cell shape and motility, hemolysis, production of catalase, oxidase, urease and lecithinase, nitrate reduction, fermentation of D-glucose, maltose, D-xylose, lactose and D-mannitol, and growth at a temperature of 10 °C. *Bacillus cereus* ATCC 11778 and *B. cereus* ATCC 14579 were used as reference strains for phenotypic tests.

### PCR detection of virulence factor genes in *B. cereus s. l.*

Prior to DNA extraction, bacterial cultures were grown by streaking on nutrient agar and incubating at 30 °C for 24 h for preparation of template DNA for PCR screening. The bacterial genomic DNA of was extracted using the InstaGene Matrix DNA extraction kit following the instructions of the manufacturer (Bio-Rad, Hercules, CA, USA).

PCR screening was done to detect the presence of 8 enterotoxigenic genes (*hblA*, *hblC*, *hblD*, *nheA*, *nheB*, *nheC*, *cytK* and *entFM*), and one emetic gene (*ces*). The primer pair sequences used for the amplification of virulence factor genes of *B. cereus* in this study are shown in Table 1. The PCR reaction was carried out as described by Kim et al. [31]. Briefly, the PCR reaction mixture contained 25 ng of template DNA, 0.5 U dreamTaq DNA polymerase (Fermentas GmbH, St. Leon-Rot, Germany), 10 mM Tris-HCl (pH 8.3), 10 mM KCl, 0.2 mM each deoxynucleoside triphosphate (dNTPs), 2.5 mM MgCl_2_, and 1 μM each primer. Sterile MilliQ water was used for the preparation of the PCR mixture and for all negative control reactions. The reaction was performed in an automatic thermal cycler (Biotron, Göttingen, Germany) under the following optimized cycling program: an initial denaturation step of 3 min at 95 °C; 35 cycles of denaturation at 94 °C for 30 s, annealing at 58 °C for 45 s, extension at 72 °C for 1.5 min; and a final extension at 72 °C for 5 min.

**Table 1:** Sequences of PCR primers targeting various targeting various virulent factor genes in this study

Target gene	Primer	Primer sequence (5′ – 3′)	Amplicon size (bp)	Reference
*nheA*	nheA 344 S	TACGCTAAGGAGGGGCA	480	[11]
	nheA 843 A	GTTTTTATTGCTTCATCGGCT		
*nheB*	nheB 1500 S	CTATCAGCACTTATGGCAG	754	[11]
	nheB 2269 A	ACTCCTAGCGGTGTTCC		
*nheC*	nheC 2820 S	CGGTAGTGATTGCTGGG	564	[11]
	nheC 3401 A	CAGCATTCGTACTTGCCAA		
*hblA*	HBLA1	GTGCAGATGTTGATGCCGAT	301	[11]
	HBLA2	ATGCCACTGCGTGGACATAT		
*hblC*	L2A	AATGGTCATCGGAACTCTAT	731	[11]
	L2B	CTCGCTGTTCTGCTGTTAAT		
*hblD*	L1A	AATCAAGAGCTGTCACGAAT	411	[11]
	L1B	CACCAATTGACCATGCTAAT		
*cytK*	CK-F-1859	ACAGATATCGG(GT)CAAAATGC	809	[46]
	CK-R-2668	TCCAACCCAGTT(AT)(GC)CAGTTC		
*entFM*	ENTA	ATGAAAAAAGTAATTTGCAGG	1, 269	[63]
	ENTB	TTAGTATGCTTTTGTGTAACC		
*Ces*	cesF1	GGTGACACATTATCATATAAGGTG	1, 271	[16]
	cesR2	GTAAGCGAACCTGTCTGTAACAACA		

The amplified PCR fragments were analysed by submerged 1.5% agarose gel electrophoresis in 1x buffer (108 g Trisbase/l, 55 g boric acid/l and 40 ml of 0.5 M EDTA, pH 8.0). Following electrophoresis, gels were stained with ethidium bromide, photographed under UV illumination. A reaction mixture without DNA template served as a general control for extraneous nucleic acid contamination. Other controls including sterile MilliQ water and template DNA were used to detect false-positive and false-negative reactions. PCR amplification and electrophoresis experiments were all carried out in triplicates.

### Antibiotics susceptibility testing

Resistance/susceptibility to antimicrobials by *B. cereus sesu lato* isolates were determined in Mueller-Hinton (MH) broth using the broth dilution method recommended by the standard criteria of the Clinical and Laboratory Standards Institute (CLSI) guide [32–35]. The 15 antibiotics including Amoxicillin, Ampicillin, Cefepime, Chloramphenicol, Ciprofloxacin, Clindamycin, Erythromycin, Gentamycin, Oxacillin, Penicillin, Quinupristin/Dalfopristin, Rifampin, Tetracycline, Trimethoprim /sulfumethoxazole and Vancomycin were each diluted in two-fold in the range of 64 to 0.015 mg/L of MH-broth. The final inoculum of *B. cereus sesu lato* suspension in the broth media were equivalent to approximately 1 × 10^3^ CFU/ml. Growth was carried out at 35 ± 2 °C for 18–20 h incubation period and examined in a microplate reader (OD at 610 nm). The breakpoints against *B. cereus* in the CLSI guideline M45A2E (2010) and M45-P (2005) were used for all antimicrobial agents except oxacillin and quinupristin/dalfopristin for which the breakpoints for *Staphylococcus* spp. in the CLSI guideline M 100-S22 (2012) and S24 (2014) were used according to Luna et al. [35].

## Results and discussion

### Prevalence and phenotypic characteristics of *Bacillus cereus s. l.*

The prevalence of *B. cereus sensu lato* in soil from cattle grazing farms, raw milk, nunu, and woagashie are shown in Table 2. Eighteen of 25 (72.0%) soil, 14 of 30 (46.6%) raw milk, 10 of 28 (35.7%) nunu and 12 of 31 (38.7%) woagashi samples were positive for *B. cereus sensu lato* with mean counts (log_10_ CFU/g) of 4.2 ± 1.8, 3.3 ± 2.0, 1.8 ± 1.4 and 2.6 ± 1.8 respectively (Table 2). All the isolates showed common phenotypic and biochemical characteristics that are consistent with the identification of *Bacillus cereus sensu lato*. The isolates were motile rods with peritrichous flagella, and haemolytic with β-haemolysis or lavender-green coloration under heavy growth which is an indication of proteolytic activity. Additionally, they produced catalase, lecithinase, and reduced nitrate. The isolates fermented D-glucose and maltose but were variable in their ability to ferment sucrose and lactose. None of the isolates fermented of D-xlyose and D-mannitol. Production of oxidase and urease was variable while there was no production of indole. All the isolates were able to grow at 10 °C. Thus the phenotypic and biochemical characteristics suggest that the pool of isolates selected did not include *B. anthracis* which is non-hemolytic, and *B. cytotoxicus* which has a minimum growth temperature of 20 °C [36]. Previous reports suggests that *B. cereus* strains isolated from dairy products adapt to environmental culture conditions [37] which might explain the ability of some *B. cereus* isolated from milk to ferment lactose. In analysing 334 samples of pasteurized milk, TeGiffel et al. [37] found that 40% of the samples were contaminated with *B. cereus*, out of which 53% of the *B. cereus* isolates could grow at 7 °C, and 20% fermented lactose which is an uncommon carbon source for *B. cereus* [37]. Similarly, 17 *B. cereus s. l.* isolated from milk and dairy products in the current work fermented lactose, whiles no isolate from soil could ferment lactose.

**Table 2:** Prevalence of *B. cereus sensu lato* in soil, raw milk and milk products

Sample type	Number of positive samples (%)	Mean count^a^ (log CFU/g)
Soils (*n* = 25)	18 (72.0)	4.2 ± 1.8
Raw milk (*n* = 30)	14 (46.7)	3.3 ± 2.0
Nunu (*n* = 28)	10 (35.7)	1.8 ± 1.4
Woagashie (*n* = 31)	12 (38.7)	2.6 ± 1.8

^a^Values are means ± standard deviations (SD)

*B. cereus* is widespread in the environment and shows great ecological diversity, enhancing their ability to contaminate many raw and finished food products including milk and milk products, although usually at low levels of ≤ 10^3^ CFU/g [38–42]. In the present study, raw milk and traditional milk products (nunu and woagashie) all had *B. cereus* counts of <4 log CFU/g. In general, it is estimated that consumption of food containing *B. cereus* cells and/or spores between 10^5^ and 10^8^ can cause disease [9, 43]. Therefore, the load of *B. cereus sensu lato* in milk and milk products in the present study are within acceptable limits for consumption according to the EFSA recommended level of <10^5^ CFU/g at the point of consumption. However, there are reported cases of both emetic and diarrhoeal diseases involving lower levels (below 10^5^ cfu/g) of *B. cereus* [43]. Therefore the potential for *B. cereus* infections through the consumption of unpasteurized milk and milk products in Ghana cannot be underestimated. Additionally, Ghana lacks proper foodborne diseases surveillance systems to provide reliable data on the burden of foodborne illnesses involving *B. cereus* in milk and milk products. Thus a number of illnesses or sporadic outbreaks of *B. cereus* infections resulting from the consumption of unpasteurized milk and milk products may go unreported. Because *B. cereus* are generally resident flora of soil and frequently associated with farm environments and the fecal shedding of cattle, there is a higher risk of contamination of milk, and subsequent entry into the dairy food chain where they can cause spoilage and/or diseases. It is therefore important for dairy farmers and processors of traditional milk products to practice high level of good hygienic practices (GHP) and Good manufacturing practices (GMP), as well as implement the use of starter cultures for the production fermented dairy products.

### Distribution of virulence factor genes among *B. cereus s. l.* isolates

**Fig. 1:**
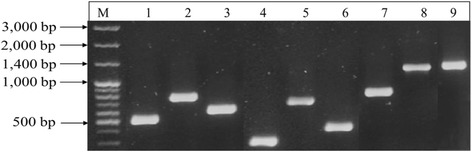
Representative PCR products detecting various virulence factor genes in *B. cereus s. l*. isolated from soil and various dairy products. Lane M, 100 bp molecular size DNA marker; lane 1, *nheA*; lane 2, *nheB*; lane 3, *nheC*; lane 4, *hblA*; lane 5, *hblC*; lane 6, *hblD*; lane 7, *cytK*; lane 8, *entFM*; lane 9, *Ces*

PCR based detection of Virulence factors in *B. cereus sensu lato* targeted genes encoding enterotoxins and emetic toxin. These included genes encoding haemolytic (*hblA*, *hblC*, and *hblD*) and non-haemolytic (*nheA*, *nheB*, and *nheC*) enterotoxin complexes, cytotoxin K (*cytK*), enterotoxin FM (*entFM*), and cereulide (*ces*) as shown in Fig. 1. All primers used produced amplicons of the expected size from their respective target virulence genes with reproducible results in repeated experiments. The distribution of virulence genes among 96 *B. cereus s. l.* isolates are shown in Table 3. For HNE encoding genes, about 60% (58/96) of *B. cereus s. l.* isolates were found to harbour simultaneously the *nheABC* genes, 13% (12/96) harboured only one gene, 19% (18/96) harboured simultaneously two genes, and 8% possessed none of the NHE encoding genes. For HBL encoding genes, 38% (37/96) possessed only one gene, 24% (23/96) possessed simultaneously two genes, 13% (12/96) possessed simultaneously all three *bhlACD* genes, and 25% (24/96) possessed no HBL encoding gene at all. The prevalence of *cytk*, *entFM*, and the emetic gene *ces* among *B. cereus s. l*. isolates were 75, 67 and 9% respectively. The emetic gene was only detected in *B. cereus s. l.* isolated from milk and milk products but not from soil samples.

**Table 3:** Distribution of enterotoxin and emetic toxin genes in *B. cereus senso lato* isolated from dairy farm and milk product

Toxigenic genes	Number of strains (%)^a^ positive for target gene(s)				
	Soil (*n* = 30)	Raw milk (*n* = 24)	Nunu (*n* = 18)	Woagashie (*n* = 24)	Total (*n* = 96)
NHE gene complexes					
*nheA*	3 (10)	1 (4)	0 (0)	2 (8)	6 (6)
*nheB*	1 (3)	1 (4)	0 (0)	0 (0)	2 (2)
*nheC*	1 (3)	0 (0)	1 (6)	2 (8)	4 (4)
*nheA* + *nheB*	2 (7)	2 (8)	0 (0)	1 (4)	5 (5)
*nheA* + *nheC*	0 (0)	0 (0)	0 (0)	0 (0)	0 (0)
*nheB* + *nheC*	2 (7)	3 (13)	4 (22)	4 (17)	13 (14)
*nheA* + *nheB* + *nheC*	19 (63)	15 (63)	9 (50)	15 (63)	58 (60)
None detected	2 (7)	2 (8)	4 (22)	0 (0)	8 (8)
HBL gene complexes					
*hblA*	4 (13)	4 (17)	1 (6)	2 (8)	11 (11)
*hblC*	1 (3)	5 (21)	3 (17)	1 (4)	10 (10)
*hblD*	5 (17)	2 (8)	2 (11)	7 (29)	16 (17)
*hblA* + *hblC*	0 (0)	3 (13)	0 (0)	0 (0)	3 (3)
*hblA* + *hblD*	6 (20)	2 (8)	1 (6)	3 (13)	12 (13)
*hblC* + *hblD*	1 (3)	3 (13)	0 (0)	4 (17)	8 (8)
*hblA* + *hblC* + *hblD*	5 (17)	1 (4)	4 (22)	2 (8)	12 (13)
None detected	8 (27)	4 (17)	7 (39)	5 (21)	24 (25)
Other genes					
*cytK*	9 (30)	21 (88)	18 (100)	24 (100)	72 (75)
*entFM*	19 (63)	17 (71)	15 (83)	13 (54)	64 (67)
*Ces*	0 (0)	5 (21)	1 (6)	3 (13)	9 (9)

^a^percentages have been converted to the nearest whole numbers

Virulence factors HBL, NHE and cytotoxin K are primarily responsible for the production of *B. cereus s. l*. enterotoxins [12, 14, 44]. The isolated *B. cereus s. l.* commonly possessed *cytk* (75%) as the most prevalent toxin gene followed by *entFM* (67%). Similar results of high prevalence of *nheABC* and *entFM* gene complexes have previously been reported to be widespread among wild *B. cereus* isolates from various food and environmental sources [30, 45–48], as well as some reference strains [31]. Similarly, various studies have reported higher prevalence rates, usually between 40 and 70.6% of the HBL gene complex in *B. cereus* isolated from milk and dairy products [28, 49–51]. The prevalence rate of the emetic toxin gene *ces* was 9%. They were however detected only in *B. cereus s. l.* isolated milk and milk product but not from soil samples. Emetic toxin producing genes have previously been detected at different low rates (1.5 to 17.2%) in isolated *B. cereus* strains isolated from various food sources [42, 48, 52]; the different prevalence rates being attributed to the differences in food property [53–55]. Kim et al., [31], did not successfully generate amplicons for the emetic gene, *ces*, in both reference and commercial strains of *B. cereus*. These findings therefore seems to suggest that emetic toxin genes are not highly prevalent or are rare among *B. cereus* isolates.

### Resistance of *B. cereus s. l*. to antibiotics

Resistance/susceptibility to different antimicrobial agents by *B. cereus s. l*. are shown in Table 4. Irrespective of the origin (soil, milk or milk products) of the isolates, they were generally resistant to ampicillin (98%), oxacillin (92%), penicillin (100%), amoxicillin (100%), cefepime (100%) and trimethoprim/sulfamethoxazole (80% with 20% intermediate resistant strains). They were however susceptible to other antimicrobials such as Chloramphenicol (99%), Ciprofloxacin (100%), Clindamycin (100%), Erythromycin (92%), Gentamicin (100%), Quinupristin/dalfopristin (100%), Rifampin (100%), Tetracycline (97%) and Vancomycin (100%).

**Table 4:** Resistance to antimicrobials by *B. cereus sensu lato* isolated from dairy farms and milk products

Antibiotic	^a^Breakpoints		^b^Interpretation *n* (%)		
	S (≤)	R (≥)	S	I	R
Amoxicillin	4	8	0	0	96 (100)
Ampicillin	0.25	0.5	0	2 (0.02)	94 (98)
Cefepime	0	0	0	0	96 (100)
Chloramphenicol	8	32	95 (99)	1 (0.01)	0
Ciprofloxacin	1	4	96 (100)	0	0
Clindamycin	0.5	4	96 (100)	0	0
Erythromycin	0.5	4	88 (92)	8 (8)	0
Gentamicin	4	16	96 (100)	0	0
Oxacillin	2	4	3 (3)	5 (5)	88 (92)
Penicillin	0.12	0.25	0	0	96 (100)
Quinupristin/dalfopristin	1	4	96 (100)	0	0
Rifampin	1	4	96 (100)	0	0
Tetracycline	4	16	93 (97)	3 (3)	0
Trimethoprim/sulfamethoxazole	2	4	0	19 (20)	77 (80)
Vancomycin	4	>4	96 (100)	0	0

^a^The breakpoints against *B. cereus* in the CLSI guideline M45A2E (2010) and M45-P (2005) were used for all antimicrobial agents except oxacillin and quinupristin/dalfopristin for which the breakpoints for *Staphylococcus* spp. in the CLSI guidelines M 100-S22 (2012) and S24 (2014) were used

^b^ *S* susceptible, *I* intermediate, *R* resistant

Because *B. cereus* have clinical significance, determining their resistance or otherwise to antimicrobial agents is critical for treatment during outbreaks. Previous reports have shown that *B. cereus* is susceptible to imipenem and vancomycin, and most strains are sensitive to chloramphenicol, aminoglycosides, ciprofloxacin, erythromycin, and gentamicin [56–59]. Some strains of *B. cereus* are moderately sensitive to clindamycin and tetracycline [58]. In this report, *B. cereus s. l.* isolated from soil from cattle grazing fields and milk and dairy products were predominantly resistant to β-lactam antibiotics. The abundant production of β-lactamases by bacteria including *Bacillus* species is a common cause of antibiotic resistance in bacteria [39, 60]. Wild-type genomes of many bacteria, including *Bacillus* species have been found to possess genes encoding the production of β-Lactamase. However, these chromosomal β-lactamases do not generally provide effective antibiotic resistance in wild-type bacilli, despite evidence that the genes are not completely silenced [61, 62].

## Conclusions

*Bacillus cereus sensu lato* is prevalent in soil from cattle grazing fields, raw milk and traditional dairy products, but load in milk and traditional dairy products are at levels considered to be safe for consumption. However, various enterotoxin genes associated with the virulence of *B. cereus* are widespread among isolates. Additionally, the *B. cereus s. l*. isolates were generally resistant to β-lactam antibiotics but susceptible to other antibiotics. There is therefore the need to observe good hygienic and manufacturing practice by milk producers and traditional dairy processors to prevent contamination and subsequent potential disease outbreak by *B. cereus*.

## Abbreviations

CLSI: Clinical and Laboratory Standards Institute
DNA: de-oxyribonucleic acid
EDTA: Ethylenediaminetetraacetic acid
EFSA: European food safety authority
GHP: Good hygienic practices
GMP: Good manufacturing practices
HBL: Hemolysin BL
MH: Muellar-Hinton
NHE: Non-hemolytic enterotoxin
PCR: Polymerase chain reaction
s.l.: *Sensu* lato
